# Exploring strong controlled partial metric type spaces: Analysis of fixed points and theoretical contributions

**DOI:** 10.1016/j.heliyon.2024.e39525

**Published:** 2024-10-18

**Authors:** Dania Santina, Wan Ainun Mior Othman, Kok Bin Wong, Nabil Mlaiki

**Affiliations:** a Department of Mathematics and Sciences, Prince Sultan University Riyadh, 11586, Saudi Arabia; b Institute of Mathematical Sciences, Faculty of Science, University Malaya, Kuala Lumpur, Malaysia

**Keywords:** 47H10, 54H25, Metric space (Ms), Fixed point (FP), Controlled metric space (CMs), Controlled partial metric type spaces, Boundary Value Problem (BVP)

## Abstract

This study involves the generalization of partial metric spaces and strong controlled metric type spaces through the introduction of the notion of strong controlled partial metric type spaces. This research pursues a dual objective: firstly, to scrutinize fixed points related to contractions of the Kannan type within the framework of strong controlled partial metric type spaces, encompassing both linear and nonlinear contractions; secondly, to furnish illustrative examples and applications that underscore the consequential implications of our theoretical contributions.

## 1 Introduction

In 1906, Maurice Fréchet introduced the foundational concept of metric spaces, which provided a framework for analyzing distances between points in a generalized setting [1]. This pioneering work laid the groundwork for subsequent developments in the field of topology and analysis. Building upon Fréchet's foundational notions, Stefan Banach established a new area of study known as fixed point theory in 1922. This branch of mathematics focuses on the conditions under which mappings on metric spaces have points that remain invariant under those mappings, significantly contributing to both theoretical and applied mathematics [2].

In the years that followed, researchers continued to expand the understanding and applications of metric spaces. Notably, in 1923, Bakhtin and Czerwik introduced the concept of b-metric spaces, further refining the framework of metric spaces by allowing for more generalized notions of distance [3] [4]. This advancement has enabled a richer exploration of the properties and behaviors of functions within these spaces, paving the way for further research and applications in various mathematical disciplines. Numerous researchers have investigated the presence of multi-valued operators with fixed points in b-metric spaces, as demonstrated in studies such as [5], [6], [7], [8], [9], [10], [11], [12], [13], [14]. Illustrating a broadening of the concept of b-metric spaces, Kamran et al. [15] introduced an expanded version wherein the constant coefficient in the triangular inequality is replaced with a function. In 2018, Abdeljawad et al. introduced the concepts of controlled metric spaces and double controlled metric spaces [16] [17] [18]. Subsequently, in 2023, the concept of controlled strong was introduced as an extension of b-metric space by substituting the constant with a function and multiplying it solely with the second addend in the triangular inequality [19].

This study explores the concept of partial metric spaces, a notable extension of traditional metric spaces that was introduced by Matthews in 1992. This innovative framework was specifically designed to address certain challenges encountered in the domain theory of computer programming [20]. A key aspect of partial metric spaces is the stipulation that the self-distance of a point is distinctly non-zero, which contrasts with the conventional definition of metric spaces where the distance from a point to itself is always zero.

In the years following Matthews' introduction of partial metric spaces, a variety of researchers have undertaken significant efforts to establish the existence and uniqueness of fixed points within this framework, as documented in the literature [21]. These investigations have not only enriched the theoretical landscape but have also highlighted the practical implications of partial metric spaces in various fields.

Building on these foundational ideas, this study aims to further develop the concepts of strong controlled metric spaces and partial metric spaces [22]. Specifically, we introduce the notion of a strong controlled partial metric space, which serves as a new framework for understanding the properties and behaviors of functions within this context. Our research places particular emphasis on establishing both the existence and uniqueness of fixed points within complete strong controlled partial metric spaces.

Each theorem presented in this article meets the necessary conditions for conducting the ratio test, ensuring the convergence of the sequences involved. In the concluding section of our study, we apply our findings to solve a fourth-order differential equation, demonstrating the practical relevance of the theoretical advancements we propose. This comprehensive approach not only contributes to the existing body of knowledge surrounding partial metric spaces but also showcases their applicability in solving complex mathematical problems.

In addition to the theoretical advancements and practical applications discussed, this study also aims to foster further exploration within the realm of partial metric spaces. By introducing the concept of strong controlled partial metric spaces, we encourage future researchers to investigate the myriad ways in which these structures can be utilized across various mathematical disciplines. Potential avenues for further research may include the exploration of more generalized forms of metric spaces, the application of our findings to other types of differential equations, or the examination of the implications of strong controlled partial metric spaces in computational theories and algorithms. Ultimately, we hope that this work will not only enhance the understanding of fixed point theory in the context of partial metric spaces but also inspire innovative applications and extensions that could benefit both theoretical research and practical implementations in diverse fields.

## 2 Preliminary claims

Initially, we review the definition of strong-controlled *b*− Ms for the observer.

Definition 2.1 Let *E* be a nonempty set and a self mapping ξ:E×E → [1,∞). The following function Ω:E×E→[0,∞) is called a strong-controlled *b*-Ms if

(1) Ω(i,j)=0 iff i=j;
(2) Ω(i,j)=Ω(j,i);
(3) Ω(i,j)≤Ω(i,k)+ξ(k,j)Ω(k,j),

for all i,j,k∈E. The pair (E,Ω) is said to be a strong-controlled *b*-Ms.

The next following description is for next, we illustrate the definition of strong-controlled Partial Ms.

Definition 2.2 Let *E* be a nonempty set and a self mapping ξ:E×E → [1,∞). The following function Ω:E×E → [0,∞) is called a strong-controlled Partial Ms if

(1) i=j if and only if Ω(i,i)=Ω(j,j)=Ω(i,j);
(2) Ω(i,j)=Ω(j,i);
(3) Ω(i,i)≤Ω(i,j)
(4) Ω(i,j)≤Ω(i,k)+ξ(k,j)Ω(k,j),

for all i,j,k∈E. The pair (E,Ω) is called a strong-controlled partial Ms.

Now, we provide a case study of a strong-controlled partial Ms that is not a strong-controlled Ms. A strong-controlled partial Ms extends further than strong-controlled Ms.

Example 2.3 Let E={0,5,6,7,8} and take Ω:E×E⟶[0,∞).

Consider ξ:E×E⟶[1,∞), where $\xi (a,b)=\Omega (a,b)+e^{7}$

Consider the following table to be a definition of the metric Ω.

Verifying the truth of (1) and (2) of Definition 2.2 is a breeze. Using a selection from Table 1 of instances, we demonstrate condition 3: $\Omega \left(a_{1},a_{1}\right)\leq \Omega \left(a_{1},a_{2}\right)$, for all $a_{1}$, $a_{2}\in E$ and $a_{1}\neq a_{2}$.

**Table 1:** Values of the metric output

Ω	0	5	6	7	8
0	2/*e*^30^	2/*e*^5^	2/*e*^6^	2/*e*^7^	2/*e*^8^
5	2/*e*^5^	2/*e*^31^	3/*e*^8^	3/*e*^10^	3/*e*^12^
6	2/*e*^6^	3/*e*^8^	2/*e*^32^	4/*e*^10^	4/*e*^12^
7	2/*e*^7^	3/*e*^10^	4/*e*^10^	2/*e*^33^	5/*e*^12^
8	2/*e*^8^	3/*e*^12^	4/*e*^12^	5/*e*^12^	2/*e*^30^

Instance (1): let $\Omega \left(a_{1},a_{1}\right)=\Omega (0,0)=(2/e^{27}),\Omega (0,0)\leq \Omega \left(a_{1},a_{2}\right)$, satisfied for all $a_{1}$, $a_{2}\in E$ and $a_{1}\neq a_{2}$.

Instance (2): let $\Omega \left(a_{1},a_{1}\right)=\Omega (5,5)=(2/e^{31}),\Omega (5,5)\leq \Omega \left(a_{1},a_{2}\right)$, satisfied for all $a_{1}$, $a_{2}\in E$ and $a_{1}\neq a_{2}$

Instance (3): let $\Omega \left(a_{1},a_{1}\right)=\Omega (6,6)=(2/e^{32}),\Omega (6,6)\leq \Omega \left(a_{1},a_{2}\right)$, satisfied for all $a_{1},a_{2}\in E$ and $a_{1}\neq a_{2}$.

Instance (4): let $\Omega \left(a_{1},a_{1}\right)=\Omega (7,7)=(2/e^{33}),\Omega (7,7)\leq \Omega \left(a_{1},a_{2}\right)$, satisfied for all $a_{1},a_{2}\in E$ and $a_{1}\neq a_{2}$

Instance (5): let $\Omega \left(a_{1},a_{1}\right)=\Omega (8,8)=(2/e^{30}),\Omega (8,8)\leq \Omega \left(a_{1},a_{2}\right)$, satisfied for all $a_{1}$, $a_{2}\in E$ and $a_{1}\neq a_{2}$

We will now demonstrate the property's validity (4):

Instance (1): Proving that Ω(0,0) is satisfied, we show:

$$\Omega (0,0)\leq \Omega (0,0)+\xi (0,0)\Omega (0,0)1.8⁎10^{-13}\leq 2⁎10^{-10}\;\Omega (0,0)\leq \Omega (0,5)+\xi (5,0)\Omega (5,0)1.8⁎10^{-13}\leq 14.791\;\Omega (0,0)\leq \Omega (0,6)+\xi (6,0)\Omega (6,0)1.8⁎10^{-13}\leq 5.44\;\Omega (0,0)\leq \Omega (0,7)+\xi (7,0)\Omega (7,0)1.8⁎10^{-13}\leq 2\;\Omega (0,0)\leq \Omega (0,8)+\xi (8,0)\Omega (8,0)1.8⁎10^{-13}\leq 0.74.$$

Instance (2): Presently, we must fulfill, Ω(0,5)=Ω(5,0):

Ω(0,5)≤Ω(0,0)+ξ(0,5)Ω(0,5)0.013≤14.778Ω(0,5)≤Ω(0,5)+ξ(5,5)Ω(5,5)0.013≤0.0134Ω(0,5)≤Ω(0,6)+ξ(6,5)Ω(6,5)0.013≤1.109Ω(0,5)≤(0,7)+ξ(7,5)Ω(7,5)0.013≤0.151Ω(0,5)≤Ω(0,8)+ξ(8,5)Ω(8,5)0.013≤0.021

Instance (3): to show Ω(0,6)=Ω(6,0)

Ω(0,6)≤Ω(0,0)+ξ(0,6)Ω(0,6)0.004≤5.437Ω(0,6)≤Ω(0,5)+ξ(5,6)Ω(5,6)0.004≤1.117Ω(0,6)≤Ω(0,6)+ξ(6,6)Ω(6,6)0.004≤0.005Ω(0,6)≤Ω(0,7)+ξ(7,6)Ω(7,6)0.004≤0.201Ω(0,6)≤Ω(0,8)+ξ(8,6)Ω(8,6)0.004≤0.028

Instance (4): To achieve Ω(0,7)=Ω(7,0), we carry out the following:

Ω(0,7)≤Ω(0,0)+ξ(0,7)Ω(0,7)0.0018≤2Ω(0,7)≤Ω(0,5)+ξ(5,7)Ω(5,7)0.0018≤0.1628Ω(0,7)≤Ω(0,6)+ξ(6,7)Ω(6,7)0.0018≤0.2041Ω(0,7)≤Ω(0,7)+ξ(7,7)Ω(7,7)0.0018≤0.0018Ω(0,7)≤Ω(0,8)+ξ(8,7)Ω(8,7)0.0018≤0.0344.

Instance (5): Currently, to fulfill Ω(0,8)=Ω(8,0):

Ω(0,8)≤Ω(0,0)+ξ(0,8)Ω(0,8)0.00067≤0.7364Ω(0,8)≤Ω(0,5)+ξ(5,8)Ω(5,8)0.00067≤0.0337Ω(0,8)≤Ω(0,6)+ξ(6,8)Ω(6,8)0.00067≤0.0319Ω(0,8)≤Ω(0,7)+ξ(7,8)Ω(7,8)0.00067≤0.0355Ω(0,8)≤Ω(0,8)+ξ(8,8)Ω(8,8)0.0006709252≤0.0006709254

Instance (6): According to Ω(5,5), we proceed as

$$\Omega (5,5)\leq \Omega (5,0)+\xi (0,5)\Omega (0,5)6⁎10^{-14}\leq 14.792\;\Omega (5,5)\leq \Omega (5,5)+\xi (5,5)\Omega (5,5)6⁎10^{-14}\leq 7⁎10^{-11}\;\Omega (5,5)\leq \Omega (5,6)+\xi (6,5)\Omega (6,5)6⁎10^{-14}\leq 1.105\;\Omega (5,5\leq \Omega (5,7)+\xi (7,5)\Omega (7,5)6⁎10^{-14}\leq 0.149\;\Omega (5,5)\leq \Omega (5,8)+\xi (5,8)\Omega (8,5)6⁎10^{-14}\leq 0.02$$

Instance (7): to fulfill Ω(5,6)=Ω(6,5), we have

Ω(5,6)≤Ω(5,0)+ξ(0,6)Ω(0,6)0.001≤5.45Ω(5,6)≤Ω(5,5)+ξ(5,6)Ω(5,6)0.001≤1.103,Ω(5,6)≤Ω(5,6)+ξ(6,6)Ω(6,6)0.0010063878≤0.0010063879,Ω(5,6)≤Ω(5,7)+ξ(7,6)Ω(7,6)0.001≤0.199,Ω(5,6)≤Ω(5,8)+ξ(8,6)Ω(8,6)0.001≤0.0269

Instance (8): Currently, to prove Ω(5,7)=Ω(3,7):

$$\Omega (5,7)\leq \Omega (5,0)+\xi (0,7)\Omega (0,7)1.3⁎10^{-4}\leq 2.0.13\;\Omega (5,7)\leq \Omega (5,5)+\xi (5,7)\Omega (5,7)1.3⁎10^{-4}\leq 0.14,\Omega (5,7)\leq \Omega (5,7)+\xi (6,7)\Omega (6,7)1.3⁎10^{-4}\leq 0.2,\Omega (5,7)\leq \Omega (5,7)+\xi (7,7)\Omega (7,7)1.3619978⁎10^{-4}\leq 1.3619979⁎10^{-4},\Omega (5,7)\leq \Omega (5,8)+\xi (8,7)\Omega (8,7)1.3⁎10^{-4}\leq 0.034$$

Instance (9): Consider the following for the situation Ω(5,8)=Ω(8,5).

$$\Omega (5,8)\leq \Omega (5,0)+\xi (0,8)\Omega (0,8)1.8⁎10^{-5}\leq 0.749,\Omega (5,8)\leq \Omega (5,5)+\xi (5,8)\Omega (5,8)1.8⁎10^{-5}\leq 0.02,\Omega (5,8)\leq \Omega (5,6)+\xi (6,8)\Omega (6,8)1.8⁎10^{-5}\leq 0.027,\Omega (5,8)\leq \Omega (5,7)+\xi (7,8)\Omega (7,8)1.8⁎10^{-5}\leq 0.034,\Omega (5,8)\leq \Omega (5,8)+\xi (8,8)\Omega (8,8)1.84326⁎10^{-5}\leq 1.84328⁎10^{-5}$$

Instance (10): Now, for Ω(6,6), we continue as follows:

$$\Omega (6,6)\leq \Omega (6,0)+\xi (0,6)\Omega (0,6)2.5⁎10^{-14}\leq 5.44,\Omega (6,6)\leq \Omega (6,5)+\xi (5,6)\Omega (5,6)2.5⁎10^{-14}\leq 1.1046,\Omega (6,6)\leq \Omega (6,6)+\xi (6,6)\Omega (6,6)2.5⁎10^{-14}\leq 2.7⁎10^{-11},\Omega (6,6)\leq \Omega (6,7)+\xi (6,7)\Omega (6,7)2.5⁎10^{-14}\leq 0.199,\Omega (6,6)\leq \Omega (6,8)+\xi (8,6)\Omega (8,6)2.5⁎10^{-14}\leq 0.027$$

Instance (11): to fulfill Ω(6,7)=Ω(7,6), we have:

$$\Omega (6,7)\leq \Omega (6,0)+\xi (0,7)\Omega (0,7)1.8⁎10^{-4}\leq 2.004\;\Omega (6,7)\leq \Omega (6,5)+\xi (5,7)\Omega (5,7)1.8⁎10^{-4}\leq 0.15\;\Omega (6,7)\leq \Omega (6,6)+\xi (6,7)\Omega (2,7)1.8⁎10^{-4}\leq 0.199\;\Omega (6,7)\leq \Omega (6,7)+\xi (7,7)\Omega (7,7)1.8159971⁎10^{-4}\leq 1.8159972⁎10^{-4}\;\Omega (6,7)\leq \Omega (6,8)+\xi (8,7)\Omega (8,7)1.8⁎10^{-4}\leq 0.0337$$

Instance (12): Then, we must fulfill Ω(6,8)=Ω(8,6):

$$\Omega (6,8)\leq \Omega (6,0)+\xi (0,8)\Omega (0,8)2.4576⁎10^{-5}\leq 0.741\;\Omega (6,8)\leq \Omega (6,5)+\xi (5,8)\Omega (5,8)2.4576⁎10^{-5}\leq 0.021\;\Omega (6,8)\leq \Omega (6,6)+\xi (6,8)\Omega (6,8)2.4576⁎10^{-5}\leq 0.0269\;\Omega (6,8)\leq \Omega (6,7)+\xi (7,8)\Omega (7,8)2.4576⁎10^{-5}\leq 0.034\;\Omega (6,8)\leq \Omega (6,8)+\xi (8,8)\Omega (8,8)2.4576⁎10^{-5}\leq 2.4577⁎10^{-5}$$

Instance (13): Now, let's consider the instance Ω(7,7):

$$\Omega (7,7)\leq \Omega (7,0)+\xi (0,7)\Omega (0,7)9⁎10^{-15}\leq 2.001\;\Omega (7,7)\leq \Omega (7,5)+\xi (5,7)\Omega (5,7)9⁎10^{-15}\leq 0.149\;\Omega (7,7)\leq \Omega (7,6)+\xi (6,7)\Omega (6,7)9⁎10^{-15}\leq 0.199\;\Omega (7,7)\leq \Omega (7,7)+\xi (7,7)\Omega (7,7)9⁎10^{-15}\leq 10^{-11}\;\Omega (7,7)\leq \Omega (7,8)+\xi (8,7)\Omega (8,7)9⁎10^{-15}\leq 0.038$$

Instance (14): Now, to prove that the condition Ω(7,8)=Ω(8,7) is satisfied, we show that

$$\Omega (7,8)\leq \Omega (7,0)+\xi (0,8)\Omega (0,8)3⁎10^{-5}\leq 0.738\;\Omega (7,8)\leq \Omega (7,5)+\xi (5,8)\Omega (5,8)3⁎10^{-5}\leq 0.02\;\Omega (7,8)\leq \Omega (7,6)+\xi (6,8)\Omega (6,8)3⁎10^{-5}\leq 0.027\;\Omega (7,8)\leq \Omega (7,7)+\xi (7,8)\Omega (7,8)3⁎10^{-5}\leq 0.033\;\Omega (7,8)\leq \Omega (7,8)+\xi (8,8)\Omega (8,8)3.07210⁎10^{-5}\leq 3.07212⁎10^{-5}$$

Instance (15): Finally, we have the following for scenario Ω(8,8):

$$\Omega (8,8)\leq \Omega (8,0)+\xi (0,8)\Omega (0,8)3⁎10^{-5}\leq 0.738\;\Omega (8,8)\leq \Omega (8,5)+\xi (5,8)\Omega (5,8)3⁎10^{-5}\leq 0.020\;\Omega (8,8)\leq \Omega (8,6)+\xi (6,8)\Omega (6,8)3⁎10^{-5}\leq 0.027\;\Omega (8,8)\leq \Omega (8,7)+\xi (7,4)\Omega (7,8)3⁎10^{-5}\leq 0.033\;\Omega (8,8)\leq \Omega (8,8)+\xi (8,8)\Omega (8,8)3.07210⁎10^{-5}\leq 3.07212⁎10^{-5}$$

As Ω(a,a) does not always equal zero, the result is that (E,Ω) is a strong-controlled partial Ms however it is not a strong-CMs.

Example 2.4 Consider E={0,1,2}. Let Ω be defined hereafter

$$\Omega (0,0)=\Omega (1,1)=\Omega (2,2)=\frac{1}{8}$$

and

$$\Omega (0,1)=\Omega (1,0)=\frac{1}{2},\;\Omega (0,2)=\Omega (2,0)=6,\;\Omega (1,2)=\Omega (2,1)=5,$$

consider $\xi :E^{2}\to [1,\infty )$ that matches itself such that

$$\xi (0,0)=\xi (1,1)=\xi (2,2)=\xi (0,2)=1,\;\xi (1,2)=\frac{5}{4},\;\xi (0,1)=\frac{11}{10}.$$

It's not too challenging to verify that (E,Ω) is a strong-controlled partial Ms, however, it is not a strong-controlled Ms. since $\Omega (2,2)=\frac{1}{8}$.

Following this, we address some topological characteristics of strong-controlled partial Ms.

Definition 2.5 Let (E,Ω) be a strong-controlled partial Ms having the sequence ${\left\{r_{n}\right\}}_{n\geq 0}$ in *E*.

(1) Convergence of the sequence $\left\{r_{n}\right\}$ to an arbitrary element *r* of set *E* is proven if and only if

$\lim_{n\to \infty }\Omega (r_{n},r)=\Omega (r_{n},r_{n})=\Omega (r,r)\;$ for all n≥N. In this instance, we shall be penning, the $\lim_{n\to \infty }r_{n}=r$.

(2) The sequence $\left\{r_{n}\right\}$ is said to be Cauchy, if $\lim_{n,m\to \infty }\Omega (r_{n},r_{m})$ exists and is finite.

(3) The strong-controlled partial Ms (E,Ω) is deemed complete if each Cauchy sequence is convergent.

Definition 2.6 Let *E* be a nonempty set and consider a self-mapping ξ:E×E→[1,∞). Let (E,Ω) denote a strong-controlled partial metric space equipped with a sequence ${\left\{r_{n}\right\}}_{n\geq 0}$ in *E*.

(1) The sequence $\left\{r_{n}\right\}$ is defined as 0-Cauchy if
  $$\lim_{n,m\to \infty }\Omega (r_{n},r_{m})=0.$$
(2) The strong-controlled partial metric space (E,Ω) is termed 0-Cauchy complete if every 0-Cauchy sequence in *E* converges to a point r∈E such that Ω(r,r)=0.

Remark 2.7

• In a strong-controlled partial metric space, every 0-Cauchy sequence is a Cauchy sequence; however, the reverse is not necessarily true.
• Every Cauchy complete strong-controlled partial metric space is also 0-Cauchy complete, but the converse does not hold in general.
• A subsequence of a 0-Cauchy sequence is also a 0-Cauchy sequence.

It is straightforward to observe that the following result holds:

Lemma 2.8 *Let*(E,Ω)*be a strong-controlled partial metric space containing the sequence*${\left\{r_{n}\right\}}_{n\geq 0}$*in E. If the sequence*$\left\{r_{n}\right\}$ 0*-converges to a point a in E, then* $\left\{r_{n}\right\}$ *is a* 0*-Cauchy sequence.*

Remark 2.9 Let (E,Ω) be a strong-controlled partial metric space. Then, (E,Ω) qualifies as a strong-controlled *b*-metric space if and only if every Cauchy sequence is also a 0-Cauchy sequence.

Definition 2.10 Let (E,Ω) be a strong-controlled partial Ms where E≠ϕ. Assume that r∈E as well as Ξ>0.

(*i*) An open ball B(r,Ξ) is

B(r,Ξ)={w∈E,Ω(r,w)−Ω(r,r)<Ξ}.

(ii) The mapping τ:E→E is said to be continuous at r∈E if for every Ξ>0, there is ϵ>0 fulfilling τ(B(r,ϵ))⊆B(τr,Ξ).

Certainly, if *τ* is continuous at *r* in the setting of strong-controlled partial Ms (E,Ω), then $r_{n}\to r$ suggests that $\tau r_{n}\to \tau r$ as n→∞.

## 3 Main results

Presently, we are ready to examine the principal finding of the Banach Contraction Principle in strong-controlled Partial Ms.

Theorem 3.1 *Suppose that*(E,Ω)*is complete controlled strong partial Ms by the function*ξ:E×E→[1,∞)*. Assume that the map τ is continuous and fulfills the following:*τ:E→E*holds*

(3.1) Ω(τq,τs)≤hΩ(q,s),

∀ q,s∈E*, and* h∈(0,1)*. For* $q_{0}\in E$*, take* $q_{n}=\tau^{n}q_{0}$*. Suppose that*

(3.2) $$\xi (q_{i+1},q_{m})<\frac{1}{h}.$$

*Moreover, for all* q∈E*, assume that*

$$\lim_{n\to \infty }\xi (q,q_{n})\;\;\text{exists and is even finite}.$$

*Hence τ has a FP which is unique.*

Proof In accordance with the theorem's assumption, consider the sequence $\left\{q_{n}=\tau^{n}q_{0}\right\}$ in *E*. By utilizing (3.1), we obtain:

$$\Omega \left(q_{n},q_{n+1}\right)\leq h^{n}\Omega \left(q_{0},q_{1}\right)$$

for every *n* in (0,∞). For the two integers n,m where n<m, We find:

$$\Omega \left(q_{n},q_{m}\right)\leq \Omega \left(q_{n},q_{n+1}\right)+\xi \left(q_{n+1},q_{m}\right)\Omega \left(q_{n+1},q_{m}\right)\leq \Omega \left(q_{n},q_{n+1}\right)+\xi \left(q_{n+1},q_{m}\right)\Omega \left(q_{n+1},q_{n+2}\right)+\xi \left(q_{n+1},q_{m}\right)\xi \left(q_{n+2},q_{m}\right)\Omega \left(q_{n+2},q_{m}\right)\leq \Omega \left(q_{n},q_{n+1}\right)+\xi \left(q_{n+1},q_{m}\right)\Omega \left(q_{n+1},q_{n+2}\right)+\xi \left(q_{n+1},q_{m}\right)\xi \left(q_{n+2},q_{m}\right)\Omega \left(q_{n+2},q_{n+3}\right)+\xi \left(q_{n+1},q_{m}\right)\xi \left(q_{n+2},q_{m}\right)\xi \left(q_{n+3},q_{m}\right)\Omega \left(q_{n+3},q_{m}\right)\leq \cdots \leq \Omega \left(q_{n},q_{n+1}\right)+\sum_{i=n+1}^{m-2}\left(\prod_{j=n+1}^{i}\xi \left(q_{j},q_{m}\right)\right)\Omega \left(q_{i},q_{i+1}\right)+\prod_{t=n+1}^{m-1}\xi \left(q_{t},q_{m}\right)\Omega \left(q_{m-1},q_{m}\right)=\Omega \left(q_{n},q_{n+1}\right)+\sum_{i=n+1}^{m-1}\left(\prod_{j=n+1}^{i}\xi \left(q_{j},q_{m}\right)\right)\Omega \left(q_{i},q_{i+1}\right)\leq \Omega \left(q_{n},q_{n+1}\right)+\sum_{i=n+1}^{m-1}\left(\prod_{j=n+1}^{i}\xi \left(q_{j},q_{m}\right)\right)h^{i}\Omega \left(q_{0},q_{1}\right)\leq \Omega \left(q_{n},q_{n+1}\right)+\sum_{i=0}^{m-1}\left(\prod_{j=0}^{i}\xi \left(q_{j},q_{m}\right)\right)h^{i}\Omega \left(q_{0},q_{1}\right)-\sum_{i=0}^{n}\left(\prod_{j=0}^{i}\xi \left(q_{j},q_{m}\right)\right)h^{i}\Omega \left(q_{0},q_{1}\right)$$

We employed ξ(q,s)≥1. Let

$$S_{q}=\sum_{i=0}^{q}\left(\prod_{j=0}^{i}\xi \left(q_{j},q_{m}\right)\right)h^{i}\Omega \left(q_{0},q_{1}\right)$$

Hence, we reach

$$\Omega \left(q_{n},q_{m}\right)\leq \Omega \left(q_{n},q_{n+1}\right)+\left(S_{m-1}-S_{n}\right)$$

Applying the ratio test to the expression $V_{i}=\left(\prod_{j=0}^{i}\xi (q_{j},q_{m})\right)h^{i}\Omega (q_{0},q_{1})$

$$\frac{V_{i+1}}{V_{i}}=\frac{\prod_{j=0}^{i+1}\xi \left(q_{j},q_{m}\right)h^{i+1}\Omega \left(q_{0},q_{1}\right)}{\prod_{j=0}^{i}\xi \left(q_{j},q_{m}\right)h^{i}\Omega \left(q_{0},q_{i}\right)}\;\text{ But }\frac{\prod_{j=0}^{i+1}\xi \left(q_{j},q_{m}\right)}{\prod_{j=0}^{i}\xi \left(q_{j},q_{m}\right)}=\xi \left(q_{i+1},q_{m}\right)\text{ then }\frac{V_{i+1}}{V_{i}}=\xi \left(q_{i+1},q_{m}\right)h\;\text{ using equation (3.2) }<\frac{1}{h}\times h<1.$$

This demonstrates the convergent character of the term. Thus, the sequence $\left\{q_{n}\right\}$ is Cauchy. This means that $\Omega (q_{n},q_{m})$ exists and is finite. Because (E,Ω) is a complete strong-controlled Ms, ∃ *r* in *E* such that

$$\lim_{n\to \infty }\Omega (q_{n},r)=\Omega (r,r).$$

Hence, since *τ* is continuous we conclude that

$$r=\lim_{n\to \infty }q_{n+1}=\lim_{n\to \infty }\tau q_{n}=\tau \lim_{n\to \infty }q_{n}=\tau \;r$$

and that r is a fixed point of *τ*.

Suppose that t belong to *E* by which τt=tandr≠t. It yields to

Ω(r,t)=Ω(τr,τt)≤hΩ(r,t).

This means that Ω(r,t)=0.

On the other hand, Ω(r,r)≤Ω(r,t) then Ω(r,r)=0.

Similarly, Ω(t,t)≤Ω(r,t) then Ω(t,t)=0.

We conclude that Ω(r,t)=Ω(r,r)=Ω(t,t). Hence r=t.

Subsequently, *τ* contains a FP *r* that is unique. □

The non-linear contraction was enhanced using a controlled function as explained by Matkowski [23], leading to an FP as shown in the following Theorem.

Theorem 3.2 *Suppose that*(E,Ω)*is a complete strong-controlled partial Ms via the function*ξ(q,s)*. Suppose that*τ:E→E*fulfills for all*q,s∈E

(3.3) Ω(τq,τs)≤μ(ϒ(q,s)),ϒ(q,s)=max⁡{Ω(q,s),Ω(q,τq),Ω(s,τs)},

*where*μ:[0,∞)→[0,∞)*and*μ(d)≥μ(c)*for all d*>*c, continuous and satisfying*$\lim_{i\to \infty }\mu^{i}(v)=0$*, such that v* >0*. In addition to, assume that* ∀ $u_{0}\in E$*, we get*

(3.4) $$\underset{m\geq 1}{\sup}\lim_{i\to \infty }\;\frac{\xi (u_{i+1},u_{m})\mu^{i+1}(\Omega (u_{0},u_{1}))}{\mu^{i}(\Omega (u_{0},u_{1}))}<1,$$

*where* $u_{n}=\tau^{n}u_{0}$*,* n=0,1,.....*. If the mapping τ under consideration is continuous, then there exists a unique FP of τ (assume it λ) in such a way for all* u∈E*, we obtain* $\tau^{n}u\to \lambda$*.*

Proof Consider $\left\{u_{n}\right\}$ and $u_{0}$ to be the same as in the notion of this theorem. If $u_{m}=u_{m+1}=\tau u_{m}$ for any randomly chosen *m*, thus, it is evident that the FP is $u_{m}$. At this point, suppose that $u_{n+1}\neq u_{n}$ for each *n*. Making use of the condition (3.3),

(3.5) $$\Omega (u_{n},u_{n+1})=\Omega (\tau u_{n-1},\tau u_{n})\leq \mu (ϒ(u_{n-1},u_{n})),$$

where clearly $ϒ(u_{n-1},u_{n})=\max\{\Omega (u_{n-1},u_{n}),\Omega (u_{n},u_{n+1})\}$. If for any arbitrary *n*, we approve that $ϒ(u_{n-1},u_{n})=\Omega (u_{n},u_{n+1})$, then utilizing (3.5) along with μ(l)<l, for each *l* greater than zero, we get

$$0<\Omega (u_{n},u_{n+1})\leq \mu (\Omega (u_{n},u_{n+1}))<\Omega (u_{n},u_{n+1}),$$

that obviously results in a contradiction. As a consequence, for each *n* it must be presented as $ϒ(u_{n-1},u_{n})=\Omega (u_{n-1},u_{n})$. Lastly, this pursues that $0<\Omega (u_{n},u_{n+1})\leq \mu (\Omega (u_{n-1},u_{n}))$. Inductively, we may prove that for each n∈[0,∞), we obtain

$$0<\Omega (u_{n},u_{n+1})\leq \mu^{n}(\Omega (u_{0},u_{1})).$$

Applying the assumption of *μ*, we can conclude that the

$$\lim_{n\to \infty }\Omega (u_{n},u_{n+1})=0.$$

To verify that $\left\{u_{n}\right\}$ is Cauchy, we apply the same approach which was employed to prove Theorem 3.1. For each m>n, we will get

(3.6) $$\Omega (q_{n},q_{m})\leq \Omega (q_{n},q_{n+1})+\sum_{i=n+1}^{m-1}\left(\prod_{j=n+1}^{i}\xi (q_{j},q_{m})\right)\;\mu^{i}\Omega (q_{0},q_{1}).$$

In line with Theorem 3.1's proof, the ratio test used to the last addend within the right-hand-side of (3.6) coupled with the condition (3.4), will show that $\left\{u_{n}\right\}$ is Cauchy. In respect to (E,Ω) that is complete, ∃ λ∈E that fulfills $\lim_{n\to \infty }\Omega (u_{n},\lambda )=0$. Then, because *τ* is continuous, we conclude

$$\lim_{n\to \infty }u_{n+1}=\lim_{n\to \infty }\tau u_{n}=\tau \lim_{n\to \infty }u_{n}=\tau \lambda .$$

Hence, *λ* is a FP of *τ*. To ensure that the FP is unique, take *w* that conforms to the definition τw=w such that *λ*≠w. By (3.3), we approach to

0<Ω(λ,w)=Ω(τλ,τw)≤μ(ϒ(λ,w))=μ(Ω(λ,w))<Ω(λ,w),

that clearly yields to a contradiction. This means that Ω(λ,w)=0.

On the other hand, Ω(λ,λ)≤Ω(λ,w) then Ω(λ,λ)=0.

Similarly, Ω(w,w)≤Ω(λ,w) then Ω(w,w)=0.

We conclude that Ω(λ,w)=Ω(λ,λ)=Ω(w,w). Hence λ=w. □

Remark 3.3 With regard to Theorem 3.2, if we take into account μ(u)=vu, v falls inside the interval (0,1), thus the (3.3) condition will have the following formula:

Ω(τq,τs)≤vmax⁡{Ω(q,s),Ω(q,τq),Ω(s,τs)}.

With respect to [24] Kannan's FP result, the following theorem describes the result.

Theorem 3.4 *Let* (E,Ω) *be a complete strong-controlled partial Ms by the continuous map* τ:E→E *in the significance of the following Kannan mapping definition:*

(3.7) Ω(τq,τs)≤c[Ω(q,τq)+Ω(s,τs)],

*for all* q,s∈E *, where* $c\in (0,\frac{1}{2})$ *. Let* $u_{0}\in E$ *, then* $u_{n}=\tau^{n}u_{0}$ *. Hence, τ has a unique FP.*

Proof Take $\left\{u_{n}=\tau u_{n-1}\right\}$ which resides to *E*. Employing (3.7), we reach

$$\Omega (u_{n},u_{n+1})=\Omega (\tau u_{n-1},\tau u_{n})\leq c[\Omega (u_{n-1},\tau u_{n-1})+\Omega (u_{n},\tau u_{n})]=c[\Omega (u_{n-1},u_{n})+\Omega (u_{n},u_{n+1})].$$

Then $\Omega (u_{n},u_{n+1})\leq \frac{c}{1-c}\Omega (u_{n-1},u_{n})$. By induction, we get

$$\Omega (u_{n},u_{n+1})\leq {\left(\frac{c}{1-c}\right)}^{n}\Omega (u_{0},u_{1}),\;\;\forall n\geq 0.$$

Taking the limit for both sides as *n* approaches infinity, we obtain that $\Omega \left(u_{n},u_{n+1}\right)$ converges to zero. Our current objective is to demonstrate that the sequence $\left\{u_{n}\right\}$ is Cauchy. With the use of the inequality (3.21),

$$\Omega \left(u_{n},u_{m}\right)=\Omega \left(\tau u_{n-1},\tau u_{m-1}\right)\leq c\left[\Omega \left(u_{n-1},\tau u_{n-1}\right)+\Omega \left(u_{m-1},\tau u_{m-1}\right)\right]\leq c\left[\Omega \left(u_{n-1},u_{n}\right)+\Omega \left(u_{m-1},u_{m}\right)\right]$$

Since $\lim_{n\to \infty }\Omega \left(u_{n},u_{n+1}\right)=0$, then

$$\lim_{n\to \infty }\Omega \left(u_{n-1},u_{n}\right)=0\;and\lim_{m\to \infty }\Omega \left(u_{m-1},u_{m}\right)=0.$$

Hence, $\lim_{n,m\to \infty }\Omega \left(u_{n},u_{m}\right)=0$.

Now, we can say that $\left\{u_{n}\right\}$ is Cauchy in the complete strong-controlled partial $MT_{s}(E,\Omega )$. Thus, ∃λ∈E as the limit of $\left\{u_{n}\right\}$ in (E,Ω). Next, we show that *λ* is a FP of *τ*.

Because *τ* is given continuous, we get

$$\lim_{n\to \infty }u_{n+1}=\lim_{n\to \infty }\tau u_{n}=\tau \lim_{n\to \infty }u_{n}=\tau \lambda .$$

Hence, *λ* is a F.P. of *τ*.

Now, we show that the FP *λ* is unique.

Presume that *τ* has two FPs *λ* and *w*. Then

Ω(λ,λ)=Ω(τλ,τλ)≤c[Ω(λ,τλ)+Ω(λ,τλ)]≤2cΩ(λ,τλ)=2cΩ(λ,λ)

where 0<2c<1. This implies that Ω(λ,λ)=0

 Similarly, Ω(w,w)=2cΩ(w,τw)≤2cΩ(w,w)

where 0<2c<1. This implies that Ω(w,w)=0

Ω(λ,w)=Ω(τλ,τw)=c[Ω(λ,τλ)+Ω(w,τw)]=c[Ω(λ,λ)+Ω(w,w)]=0 Then Ω(λ,λ)=Ω(w,w)=Ω(λ,w)

and we conclude that λ=w.

Hence, *τ* has a unique FP. □

## 4 Application: existence of a solution for a differential equation of the fourth order

We will examine the following issue:

$$\left\{ \begin{array}{c} {ϒ}^{4}(q)=\Omega (q,ϒ(q),{ϒ}^{\prime },{ϒ}^{\prime \prime },{ϒ}^{\prime \prime \prime })\\ ϒ(0)={ϒ}^{\prime }(0)={ϒ}^{\prime \prime }(1)={ϒ}^{\prime \prime \prime }(1)=0;\;h\in [0,1] \end{array}\right.$$

such that $J:[0,1]\times R^{3}\times R\to R$ is continuous function. Among the various applications of the boundary value problem (BVP) is the modeling of elastic beam deformations at equilibrium, where one endpoint is free and the other is fixed. Cantilever beam equation refers to a particular BVP in mechanics. Owing to their significance in mathematics, answers to such problems are crucial. With this insight, we will demonstrate that this BVP has a solution using the FP approach.

Now, consider E=C[0,1] the set of all continuous functions described in the interval [0,1] and describe the strong-controlled partial Ms by $\Omega (F,J)=\max_{p\in [0,1]}|F(p)-J(p){|}^{2}$, with $\xi :E^{2}\to [1,\infty )$ by $\xi (u_{1},u_{2})=5u_{1}+5u_{2}+3$.

Then, We investigate the BVP for the given fourth-order ordinary differential equation. Note that, we can convert the BVP problem to the following integral equation:

$$ϒ(q)=\int_{0}^{1}G(q,p)J(p,ϒ(p),{ϒ}^{\prime }(p))dp,\;\;ϒ\in C[0,1]$$

such that G(q,p) is Green's function of the homogeneous linear problem ${ϒ}^{4}(q)=0,ϒ(0)={ϒ}^{\prime }(0)={ϒ}^{\prime \prime }(1)={ϒ}^{\prime \prime \prime }(1)=0$, that is provided by

$$G(q,p)=\left\{ \begin{array}{c} \frac{1}{6}q^{2}(3p-q),\;\;0\leq q\leq p\leq 1\\ \frac{1}{6}p^{2}(3q-p),\;\;0\leq p\leq q\leq 1 \end{array}\right.$$

From (3.2), we can easily check that G(q,p) has the following properties $\frac{1}{3}q^{2}p^{2}\leq G(q,p)\leq \frac{1}{2}q^{2}\;(\text{or}\;\frac{1}{2}p^{2}),\;q,p\in [0,1]$.

Theorem 4.1 *Consider the below hypotheses to be true.*

(1) $J:[0,1]\times R^{3}\times R\to R$*is continuous.*
(2) ∃ τ∈[1,∞) *that fulfills the below axiom* ∀ ϒ,w∈E*.*
  $$\left|J(p,ϒ,{ϒ}^{\prime })-J(p,w,w^{\prime })|\leq \frac{1}{\sqrt{5}}|ϒ(p)-w(p)|,\;\;p\in [0,1\right]$$
(3) ∃ ${ϒ}_{0}\in X$ *such that for all* q∈[0,1]*, we deduce*
  $${ϒ}_{0}q\leq \int_{0}^{1}G(q,p)J(p,{ϒ}_{0}(p),{ϒ}_{0}^{\prime }(p))dp$$

*Therefore, the BVP has a solution in E.*

Proof Consider the F:E→E where

$$F(ϒ)(q)=\int_{0}^{1}G(q,p)J(p,ϒ(p),{ϒ}^{\prime }(p))dp.$$

Hence ϒ=Fϒ, resulting in BVP having only one solution.

Consider,

$$\left|F(ϒ)(q)-F(w)(q){|}^{2}=|\int_{0}^{1}G(q,p)J(p,ϒ(p),{ϒ}^{\prime }(p))dp-\int_{0}^{1}G(q,p)g(p,w(p),w^{\prime }(p))dp{|}^{2}\leq \int_{0}^{1}{\left(G(q,p)\right)}^{2}|J(p,ϒ(p),{ϒ}^{\prime }(p))-J(p,w(p),w^{\prime }(p){|}^{2}dp\leq \int_{0}^{1}\frac{1}{4}p^{4}\frac{1}{5}|ϒ(p)-w(p){|}^{2}dp\leq \frac{1}{5}\Omega (ϒ,w)\int_{0}^{1}\frac{1}{4}p^{4}dp\leq \frac{1}{5}\Omega (ϒ,w\right)$$

Therefore,

$$\Omega (F(ϒ),F(w))\leq \frac{1}{5}\Omega (ϒ,w)$$

Since all axioms of Theorem 3.1, are held, *F* has a FP and in other words, there is an *E* solution to the BVP. 

## 5 Conclusion

In this study, we have introduced and elaborated on the concept of strong-controlled partial metric spaces, effectively extending and generalizing previous notions related to strong-controlled metric spaces and partial metric spaces. Our findings serve to consolidate and expand upon the existing literature in this area, providing a robust framework for understanding the properties and behaviors of self-mappings within these spaces.

Through our analysis, we demonstrated that fixed points exist uniquely for self-mappings that satisfy both linear and nonlinear contraction conditions in strong-controlled partial metric spaces. This significant result not only reinforces the theoretical foundations of fixed point theory but also highlights its practical applicability. To illustrate the effectiveness of our approach, we presented several exemplary cases and applications, which underscore the potential for further exploration and application of these concepts in various mathematical and computational contexts.

As we conclude this work, we would like to invite the reader to consider an intriguing open question: Is it possible to establish similar results for strong-controlled partial metric spaces that are endowed with a graph structure? Addressing this question could open new avenues for research, potentially leading to a deeper understanding of the interplay between graph theory and metric space properties.

Looking ahead, we suggest several directions for future work. Researchers might investigate the implications of our findings on broader classes of metric spaces, such as those incorporating additional algebraic or topological structures. Furthermore, exploring the relationships between strong-controlled partial metric spaces and other forms of generalized metric spaces could yield insightful results. Finally, it would be beneficial to examine the application of these concepts in real-world scenarios, particularly in fields such as computer science, where the dynamics of self-mapping and fixed points play a crucial role in algorithm design and analysis. By pursuing these avenues, we can further enrich the study of strong-controlled partial metric spaces and their myriad applications.

## CRediT authorship contribution statement

**Dania Santina:** Writing – original draft. **Wan Ainun Mior Othman:** Writing – original draft. **Kok Bin Wong:** Writing – original draft. **Nabil Mlaiki:** Writing – original draft.

## Declaration of Competing Interest

The authors declare that they have no known competing financial interests or personal relationships that could have appeared to influence the work reported in this paper.

## Data Availability

No data was used for the research described in the article.
